# On the potential for extinction by Muller's Ratchet in *Caenorhabditis elegans*

**DOI:** 10.1186/1471-2148-8-125

**Published:** 2008-04-30

**Authors:** Laurence Loewe, Asher D Cutter

**Affiliations:** 1 Institute of Evolutionary Biology, School of Biological Sciences, University of Edinburgh, Edinburgh, EH9 3JT, UK; 2 Center for Systems Biology Edinburgh, School of Biological Sciences, University of Edinburgh, Edinburgh, EH9 3JU, UK; 3 Department of Ecology and Evolutionary Biology, University of Toronto, Toronto, M5S 1L2, Canada

## Abstract

**Background:**

The self-fertile hermaphrodite worm *C. elegans *is an important model organism for biology, yet little is known about the origin and persistence of the self-fertilizing mode of reproduction in this lineage. Recent work has demonstrated an extraordinary degree of selfing combined with a high deleterious mutation rate in contemporary populations. These observations raise the question as to whether the mutation load might rise to such a degree as to eventually threaten the species with extinction. The potential for such a process to occur would inform our understanding of the time since the origin of self-fertilization in *C. elegans *history.

**Results:**

To address this issue, here we quantify the rate of fitness decline expected to occur via Muller's ratchet for a purely selfing population, using both analytical approximations and globally distributed individual-based simulations from the evolution@home system to compute the rate of deleterious mutation accumulation. Using the best available estimates for parameters of how *C. elegans *evolves, we conclude that pure selfing can persist for only short evolutionary intervals, and is expected to lead to extinction within thousands of years for a plausible portion of parameter space. Credible lower-bound estimates of nuclear mutation rates do not extend the expected time to extinction much beyond a million years.

**Conclusion:**

Thus we conclude that either the extreme self-fertilization implied by current patterns of genetic variation in *C. elegans *arose relatively recently or that low levels of outcrossing and other factors are key to the persistence of *C. elegans *into the present day. We also discuss results for the mitochondrial genome and the implications for *C. briggsae*, a close relative that made the transition to selfing independently of *C. elegans*.

## Background

The bactivorous nematode *Caenorhabditis elegans *is an established model for molecular genetics, development, neurobiology, and, more recently, for evolutionary biology [1]. One of the principal features that distinguishes this species from most of its congeners is the existence of self-fertile hermaphrodites. It is now clear that hermaphrodites evolved from females in at least two *Caenorhabditis *lineages [2,3]. However, the age of self-fertilization in *C. elegans*' history is a longstanding question. The lack of a good fossil record for nematodes means that we must focus on theoretical and molecular methods for inferring the timing of such ancestral events. It is important to acquire a better understanding of how long hermaphrodites have persisted in the *C. elegans *lineage because a recent versus ancient origin of selfing will strongly influence our inferences from comparative analyses and population genetic patterns, and our interpretations about the adaptive nature of phenotypes. In the present study, we explore this issue by considering the potential for extinction to occur by way of Muller's Ratchet [4-6] in the context of parameters that describe how *C. elegans *evolves.

Muller's ratchet leads to the stochastic accumulation of slightly deleterious mutations in finite asexual populations [4,5](for a review see [6]). This process operates by the sequential loss of the class of individuals in a population that have the highest fitness, resulting in an irrecoverable ratcheting up of the mutation load. In the absence of mitigating factors, this process can lead to population extinction [7-9]. Muller's ratchet also accumulates deleterious mutations in selfers, with the dynamics being described appropriately by a simple rescaling of parameters relative to the asexual case [10-12]. This rescaling allows us to predict the rate of mutation accumulation from Muller's ratchet in selfing organisms by using a methodology established for asexual systems [6]. Consequently, we apply this approach to infer the expected time to extinction by Muller's ratchet for lines of *C. elegans *under the assumption that outcrossing stopped immediately upon the origin of the self-fertile lineage. Estimates of the effective population size for *C. elegans *based on genetic variation are generally small [13-16], particularly as compared to related obligately outbreeding species [17]; this observation reinforces the potential for Muller's ratchet to operate in this system. Because *C. elegans *populations do undergo low levels of outcrossing in nature, which could ameliorate the effects of Muller's ratchet, this approach provides a benchmark for the time over which *C. elegans *can persist in a purely self-fertile state.

Two qualitative outcomes may result from such an analysis. (i) The expected extinction times might exceed the time since divergence from the closest outcrossing relative. In this case, we could conclude that Muller's ratchet is an unimportant factor for the persistence of the species subject to selfing, because insufficient time would have elapsed to have resulted in extinction by this process. (ii) The expected time to extinction might be much shorter than the time since divergence from the nearest outcrossing relative. In this case, extinction times provide either an upper limit on the time since the origin of selfing or they indicate that some other biological process, such as outcrossing or compensatory mutation, must occur with sufficient frequency to offset genomic decay in the long term. While a variety of such potential processes are possible (see review in[6]), simpler models are preferable in the absence of evidence supporting their operation.

Here we aim to compute the most plausible estimates for the time to extinction for a lineage of *C. elegans *that reproduces purely by self-ferilization, based on the standard model of Muller's ratchet described elsewhere [12,6]. We find that, for a wide range of biologically realistic parameters, Muller's ratchet would have led to extinction in the known time of existence of the lineage leading to *C. elegans*. Several explanations could reconcile this result with the persistence of this species: (i) nearly complete selfing is a relatively recent innovation in the *C. elegans *lineage, (ii) a low level of regular outcrossing has been crucial for deleterious mutation elimination in this species history, (iii) outcrossing activity might be concentrated in a few populations that repeatedly give rise to many purely selfing lines that are then distributed around the world, (iv) adaptive or compensatory mutations repair most of the mutational damage, or (v) mutation rates in the wild are much lower than indicated by current evidence. We argue that scenario (i) is most plausible, and therefore propose that the present-day extreme form of self-fertilization seen in this species is likely to have originated relatively recently in evolutionary time, perhaps facilitated or exacerbated by the loss of pheromone attraction between the sexes [18]. However, it remains a formal possibility that low levels of outcrossing, perhaps in combination with other factors, might also play a role in the persistence of self-fertile *C. elegans *populations.

## Results

We estimated the time to extinction of a purely selfing population due to the accumulation of deleterious mutations via Muller's ratchet, based on parameter values for *C. elegans*. The genomic deleterious mutation rate, *U*_*SDM*_, is the key parameter, which we obtained by scaling estimates of the total genomic mutation rate, *U*_*TOT *_by *f*_*SDM*_, the fraction of slightly deleterious mutations. From *U*_*SDM *_we infer the rate of deleterious mutation accumulation and extrapolate it to the expected extinction times using estimates of *C. elegans *reproductive capacity and generation time. To infer the rate of mutation accumulation we use analytical approximations [19-21,6], global computing simulations [6,22] and an appropriate scaling of the key parameters to accommodate a distribution of mutational effects [6,12]. In order to account for the fact that *C. elegans *is selfing and not truly asexual, we also applied a simple transformation to double the magnitude of heterozygous mutational effects, although this has little consequence for our conclusions [12] as the distribution of mutational effects is very wide on a log-scale [23,24]. To visualize the results, we use the U-shaped plot of extinction time as a function of the selection coefficient against deleterious mutations (*s*), to characterize the range of critical selection coefficients *s*_*c *_between which extinction is expected to occur in a given interval of time [6,9]. This critical range of selection coefficients corresponds to the class of deleterious mutations that are sufficiently weak that they can accumulate, yet are strong enough to negatively impact fitness.

### Nuclear genome

**Figure 1:**
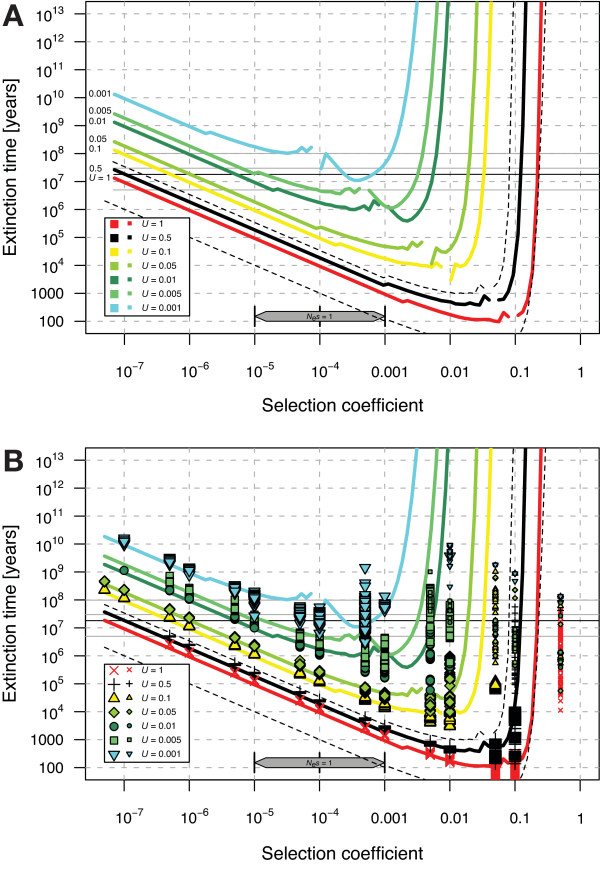
**Predicted extinction times of *C. elegans *based on mutations in the nuclear genome**. (A) Analytical results only. (B) Analytical and simulation results combined. The solid, black horizontal line denotes the estimated divergence time of *C. elegans *relative to its closest known outcrossing relatives (18 Myr), including upper and lower limits (grey lines; the upper limit of about 100 Myr from some older studies is marked separately). Extinction time estimates below this line indicate that extinction by Muller's ratchet is expected to have occurred, under a scenario of pure self-fertilization since divergence from known outcrossing sister taxa. The bar along the bottom labeled *N*_*e*_*s *= 1 indicates the boundary for selective neutrality of mutational effects (for the range of *N*_*e *_given in Table 1). Thick colored lines represent the analytic predictions of the extinction time for different effective deleterious genomic mutation rates (*U*_*sdm*_) for *N*_*e *_= 10 000, *T*_*gen *_= 60 d, and *R*_*max *_= 280 offspring/generation. Thin dashed lines demarcate bounds of uncertainty for *U*_*sdm *_= 0.5, based on upper and lower limits of *N*_*e*_, *T*_*gen *_and *R*_*max *_(Table 1); variability in extinction time is similar for other *U*_*sdm*_. Large symbols denote valid extinction time estimates from independent simulation runs with two or more observed clicks of Muller's ratchet. Small symbols denote lower limits for extinction times from simulations without observed clicks, assuming that the ratchet would have clicked just after stopping the simulation. This plot contains 36 393 simulations with a total of 19.9 years of computing time. The nearly vertical right portion of the extinction time curves represents the "wall of background selection", indicating that mutations with larger effects are eliminated deterministically.

For the nuclear genome, the results show that *C. elegans *cannot survive pure selfing for extended periods of time in the absence of mitigating forces, across most plausible parameter combinations (Figure 1). For example, considering *U*_*SDM *_= 0.5, a value that might closely reflect the true haploid genomic deleterious mutation rate (see Methods), we expect an extinction time of less than 10 Kyr (~6 × 10^4 ^generations, assuming an average 60 day generation time in nature) for a range of critical selection coefficients *s*_c _between about 0.0003 and 0.09. Even in the absence of precise estimates of the distribution of deleterious mutational effects on fitness (DDME) in *C. elegans*, we can be certain that a substantial fraction of all deleterious mutations will have effects in that range [23,24]. This assumption is confirmed by our point estimate of the DDME (see below), which suggests that *f*_*SDM *_= 51% of all mutations have effects between *s *= 0.0003 and 0.09. Generation time exerts a linear effect on the expected extinction time, so the contribution of uncertainty in this life history character does not qualitatively alter the principal conclusion (Figure 1). Maximal reproductive output *R*_*max *_enters only as its log in the computation of extinction time. Consequently, even large changes to *R*_*max *_cause only minimal changes in extinction time, rendering our results robust to this parameter, as well. The effective population size *N*_*e *_can have a large effect on the operation of the ratchet, but only for the minority of mutations with effects that are very close to the 'wall of background selection' and would be eliminated deterministically by selection; because we simulate the whole range of realistic *N*_*e *_values, our results are robust in this regard as well. In general, the conclusion that *C. elegans *cannot persist over extended periods of evolutionary time in a purely selfing state is robust to uncertainty in *s *and other parameters.

### Mitochondrial genome

**Figure 2:**
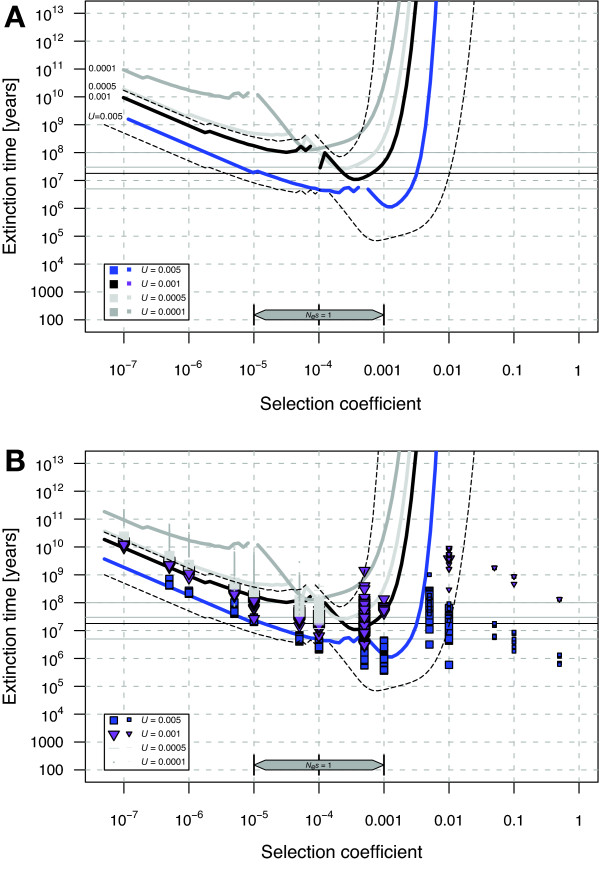
**Predicted extinction times of *C. elegans *based on mutations in the mitochondrial genome**. (A) Analytical results only. (B) Analytical and simulation results combined. See Figure 1 legend for details. Lower genomic deleterious mutation rates are chosen to reflect estimates for the mitochondrial genome (Table 1). Thin dashed lines indicate the uncertainty of the extinction time estimates for *U*_*sdm *_= 0.001 using the corresponding upper and lower limits of *N*_*e*_, *T*_*gen *_and *R*_*max*_. This plot contains 38 644 simulations with a total of 21.4 years of computing time.

The results for the mitochondrial genome in *C. elegans *are less dire (Figure 2). The parameter values that are most plausible yield extinction times close to the estimated age of common ancestry with related species (~18 Myr). Thus, the genomic decay of *C. elegans *mitochondria is not strongly implicated as an important factor limiting the persistence of this species since its divergence with sister taxa. Although we know of no evidence for or against the presence of mitochondrial recombination in *C. elegans*, despite examples from other nematodes [25,26], such a phenomenon would only reduce the potential for Muller's ratchet to operate in mitochondria.

## Discussion

These results indicate either that a predominantly selfing mode of reproduction is a recent innovation in the *C. elegans *lineage or that compensatory mutation and/or regular bouts of outcrossing contribute to species persistence. For the most plausible estimates of the nuclear deleterious mutation rate we conclude that Muller's ratchet would have led to extinction within thousands of years (tens or hundreds of thousands of generations). More generally, current knowledge about mutation rates and the distribution of deleterious mutational effects (DDME) cannot be reconciled easily with extinction times of more than a million years in the absence of outcrossing.

### Segregation in selfers

Previous work has shown that the segregation of deleterious mutations that occurs during meiosis can reduce the rate of mutation accumulation [27]. The same is true for asexual species that experience mitotic recombination [28,12]. Because recombination can stop Muller's ratchet [11,29], one might argue that segregation in selfers could be sufficient to halt Muller's ratchet. However, existing theory shows that this would be true only if all mutations have effects of a specific size such that their doubling will shift them into the domain of background selection and thus prevent their accumulation [10-12]. This is not likely to stop Muller's ratchet if there is a broad, continuous distribution of deleterious mutational effects, as is most compatible with our present understanding. If our analysis is corrected for the maximal recombinational repair that can come from free recombination within a selfer [10-12], then it still indicates that Muller's ratchet continues to operate (to apply this correction we use the haploid genomic mutation rate of *U*_*SDM *_= 0.5 instead of the diploid rate of *U*_*SDM *_= 1 in Figure 1). Thus purging of deleterious mutations by segregation in selfers is not expected to stop Muller's ratchet here.

### Outcrossing

Our analysis of Muller's ratchet assumes pure selfing, yet population genetic studies have demonstrated that outcrossing does occur at low levels in *C. elegans *[13,30,15,32]. However, recent evidence of outbreeding depression in *C. elegans *[33] and of changes in multilocus haplotype frequencies over time in nature [30], in which recombinants appear to suffer a fitness disadvantage, indicate that outcrossing and effective recombination are selected against, even if they occur within the same population. In laboratory populations, males and outcrossing are selected against, although elevated mutation rates and some genetic backgrounds partially mitigate this effect [34-38]. Furthermore, females of obligate outcrossing sister species have a pheromone that is attractive to males of all related species, but this has been lost in the selfing species *C. elegans *and *C. briggsae *[18], probably impeding the potential for male *C. elegans *to successfully locate and inseminate receptive mates in nature. Although there is evidence of some form of attraction of males to hermaphrodite-produced compounds in *C. elegans *[39,40], it would appear to be substantially weaker than in gonochoristic species [18]. Hermaphrodite *C. elegans *are less likely to mate if self-sperm is present in their reproductive tract [41]. Hermaphrodite *C. elegans *and *C. briggsae *also fail to exhibit the mate searching exhibited by females of related species [42,43] and hermaphrodites lack the mating-immobility behaviour that is observed in females of obligate outcrossing species [44], which will further obstruct successful insemination by males in nature.

These observations inform the potential for alternative causes of population persistence to occur in the face of deleterious mutation accumulation by Muller's ratchet: Is selfing a recent innovation, and rare outcrossing irrelevant to reducing the mutation load? Or, are the low levels of outcrossing sufficient to prevent extinction even over long evolutionary periods of time? Patterns of molecular evolution indicate that the reduction of effective population size observed for selfing Caenorhabditis species is unlikely to have occurred too distantly in the past [45]. The multiple genetic, behavioural, and physiological factors that reinforce selfing behaviour would also appear to favor a recent origin of extreme selfing in *C. elegans*, but a role for outcrossing in species persistence cannot be ruled out at this point. We also note that a relatively recent origin for extreme self-fertilization does not preclude a period of more moderate selfing rates in the history of breeding system evolution in the lineage leading to *C. elegans*. However, the large number of factors that reinforce selfing reproduction argue against a simple common transition from frequent outcrossing to highly selfing. Thus it appears unlikely that a few outcrossing source populations of *C. elegans *ensure long-term species persistence by continuously giving rise to many selfing lines that are doomed to extinction once they stop outcrossing regularly.

Some degree of outcrossing in self-fertile species can be sufficient to stop deleterious mutation accumulation from Muller's ratchet [11,29]. However, existing simulation results suggest that outcrossing rates of less than 1% do not reduce the rate of mutation accumulation substantially [11,29]. Thus, further simulations of occasional outcrossing in *C. elegans *might not yield conclusions that differ strongly from our results. Nevertheless, it is an important, albeit non-trivial, next step to quantitatively assess the potential impact of rare outcrossing events on expected extinction times, especially in combination with other potentially mitigating factors like advantageous mutation.

### Distribution of deleterious mutational effects (DDME)

**Figure 3:**
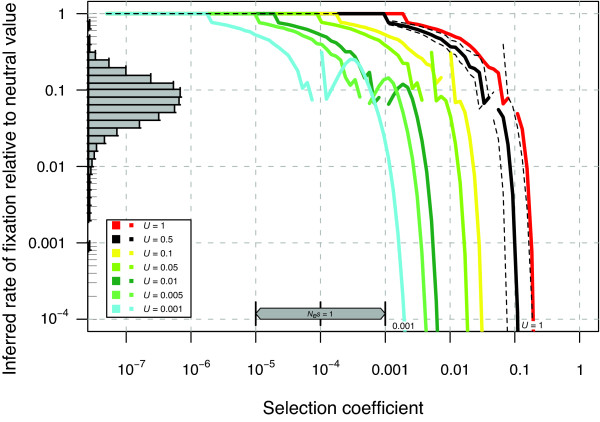
**Predicted reduction in divergence rates at sites that are under selection in nuclear genes of *C. elegans *due to Muller's ratchet**. The black dashed lines indicate the effects of variability in *N*_*e *_(1000 – 100 000). The histogram shows observed *K*_*A*_/*K*_*S *_values between *C. elegans *and *C. briggsae*, suggesting that observed divergence is roughly compatible with most selection coefficients having effects between about 0.1 and 0.0001. The inferred rate of fixation of deleterious mutations relative to the rate for neutral mutations is computed by dividing 1/*U*_*sdm *_by the predicted effective click time (parameters as in Figure 1).

For our analysis we only consider the probability mass of mutational effects in the critical range, *f*_*SDM *_(i.e. selection coefficients are in the order(s) of magnitude where extinction times are critical, see Methods). This approach is independent of any particular distribution and can be easily adapted to new findings. At the beginning of this study we had no direct estimates of the DDME in *C. elegans*. Thus we assumed that a broad range of fitness effects are introduced by mutation, as found for *Drosophila *and many other taxa [23,46,24]. The DDME should also partly be reflected in the distribution of protein divergence values as a result of variable evolutionary constraint among loci (see the nonsynonymous to synonymous divergence ratio *K*_*A*_/*K*_*S *_[47]). The wide distribution of *K*_*A*_/*K*_*S *_values between *C. elegans *and *C. briggsae *suggests an equally wide DDME (Figure 3), assuming equal mutational effects within a gene (variable intra-locus mutational effects will cause the true DDME to be even more wide). The results of Estes et al. [48] also suggest that a class of mutations exists with deleterious effects of such a size that they are not efficiently purged, and could accumulate over time [49]. Thus, our approach appears sensible in assuming a broad, continuous distribution of mutational effects.

**Figure 4:**
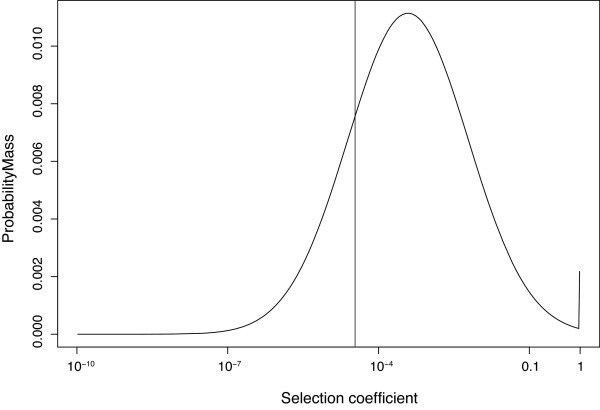
**A point estimate for the distribution of deleterious mutational effects on fitness in *Caenorhabditis***. This point estimate was computed from comparing diversity data from *C. elegans *and *C. remanei *assuming a lognormal DDME. The fraction of effectively dominant lethal mutations estimated from this distribution is biologically plausible and indicated by the spike at *s *= 1. The vertical line denotes the border to effective neutrality for *C. elegans *at *N*_*e*_*s *= 0.5. See Methods for details.

The assumption of a very wide DDME is supported by point estimates of the DDME in *C. elegans *that were obtained after completing the main part of our analysis. We used a recently developed method to estimate the DDME from nucleotide diversity data of two species (*C. elegans *and *C. remanei*) that exhibit strikingly different *N*_*e *_[23,50]. This method was applied to 730 Kbp of shotgun sequencing-based diversity data from four wild strains of *C. elegans *that were compared to the genome sequence of Bristol N2 [51] and diversity data from 40 X-linked loci in *C. remanei *[52]. The point estimate for the resulting DDME can be seen in Figure 4. It predicts that *f*_*SDM *_= 32% of all non-synonymous mutations will have effects between *s *= 0.001 and 0.05. Additional work is required to verify the robustness of this point estimate of the *Caenorhabditis *DDME.

If one were to assume that the DDME of insertions and deletions would result in their deterministic removal by purifying selection, then limiting *U *to the single nucleotide mutation rate (*U*_*SDM*_~0.2 vs. 0.5) would result in a somewhat longer expected time to extinction. We would nevertheless still expect extinction of a purely selfing lineage within hundreds of thousands of generations (Figure 1). However, the observation of substantial copy number variation in *C. elegans *[53] suggests that it is probably most appropriate to apply a mutation rate calculation that includes indels, as we have done.

Because Muller's ratchet leads to a higher rate of mutation accumulation in selfers compared to outcrossers, one might expect such a signature in the form of increased *K*_*A*_/*K*_*S *_values in *C. elegans*, when compared to outcrossing sister species [54]. However, such a signature does not appear to robustly describe a selfing versus outcrossing dichotomy [55-57]. This could result from a lack of statistical power, because divergence at effectively neutral and very strongly selected sites will be independent of selfing rates, so only a fraction of sites would be affected. However, the absence of such a signature could also indicate that selfing has a sufficiently recent origin that not enough time has elapsed to accumulate detectable sequence differences.

### Other factors

Even if *C. elegans *did not experience any outcrossing, other processes might prolong its survival (see the more comprehensive list in [6]). Several factors warrant further attention.

#### Lower mutation rates

Many factors influence mutation rates [58]. Although the evidence for the frequency of slightly deleterious mutations is quite robust compared to that in other species, it is difficult to exclude categorically the possibility of lower mutation rates in nature than inferred from the laboratory. However, the similar rates of fitness decline under mutation accumulation for different strains of *C. elegans *suggest that average mutation rates do not differ greatly among strains with low transposable element loads [59]. Furthermore, one environmental variable, temperature, does not appear to alter mutation rates substantially in this species [60].

#### Compensatory and advantageous mutations

Mutation accumulation experiments have found that compensatory mutations arise in *C. elegans *lines with compromised fitness and that they can increase fitness rapidly [61]. This kind of mutation can effectively compensate for fitness decay from Muller's ratchet [62] and might be based on variability in quantitative trait loci (QTL) that are not mechanistically related to the mutational damage that they compensate for. If long-term mutation accumulation also degrades the ability for fitness increases at these QTLs, then our results still apply (see also discussion of back mutations in [6]). Further work is necessary to elucidate the nature of compensatory mutations in *C. elegans *and a recent study demonstrated that this is possible [63]. Advantageous mutations also are powerful in stopping Muller's ratchet [64] and these cannot be distinguished from compensatory mutations in a genome that already has experienced a substantial amount of decay. Experimental evolution in viruses indicates that the beneficial mutation rate can increase as fitness declines [65], which could forestall extinction. However, it remains to be seen whether such a phenomenon is general and could apply to eukaryotes such as *C. elegans*.

#### Population structure

Natural populations of *C. elegans *show strong structure [13,14,16,32], and metapopulation processes could be important for shaping diversity. Here we assume that these effects are accounted for by using an appropriately scaled effective population size *N*_*e *_that corrects for deviations from panmixis. There is reason to believe that Muller's ratchet basically depends on *N*_*e *_and that other details of population structure can be neglected [66]. Thus our conclusions should not be strongly affected by the presence of structured populations *per se *in *C. elegans*. One potential means by which population structure could limit Muller's ratchet is if different demes experience very different outcrossing rates, leading to differential persistence of highly outcrossing demes. Inference from heterozygosity in different samples is suggestive of variable outcrossing rates [15,14,30], although both local and global patterns of polymorphism and linkage disequilibrium do not support high outcrossing rates over the long term in this species [13]. The fact that several features of *C. elegans *biology are specific to the selfing life-style (see above) further argues against the notion that there might be a long-term core of outcrossing populations that facilitate the survival of the species and that constantly produce the selfing lines that are so readily observed. In addition, theory suggests that effective outcrossing rates of less than 1% do not drastically reduce the rate of mutation accumulation [11,29], thus limiting the potential for transient instances of elevated outcrossing to impact population persistence.

### Uncertainty in divergence dates

In concluding that extinction by Muller's ratchet under pure selfing would occur in less time than has elapsed since the common ancestor of *C. elegans *and its relatives, we applied the divergence time estimates of Cutter [67]. These date estimates are 4- to 6-fold more recent than previous divergence time estimates that assumed a universal molecular clock among mammals, insects, and nematodes [68,69], and consequently are conservative for our analysis with respect to identifying a genomic decay paradox. Longer divergence times between species make it even less likely that *C. elegans *could have persisted in a purely selfing state for most of the time since divergence with the *Caenorhabditis *common ancestor.

### Mitochondrial DNA

We find that Muller's ratchet in mitochondrial DNA operates much slower than deleterious mutation accumulation in nuclear DNA, despite a higher per site mutation rate and taking into account differences in mode of transmission. This is mainly due to the much smaller mutational target of the mitochondrial genome. As a consequence, mutation load of the mitochondrial genome is not expected to be the limiting factor in species persistence for *C. elegans*. This finding may seem surprising in the light of recent results that found a substantial rate of fitness decay in human mtDNA [6]. However, (i) the shorter extinction times in human mtDNA are mostly due to higher mutation rates, which are probably a consequence of longer generation times, (ii) comparing the same mutation rates shows that absolute nematode extinction times are a bit shorter, as expected from the shorter generation time that makes *C. elegans *reach the number of generations to extinction quicker, (iii) the overwhelmingly faster speed of genomic decay in nuclear DNA, rather than an absence of decay in mtDNA, makes the mutational load in the nucleus the limiting factor. This conclusion is also robust to the possibility of stronger selection in mtDNA than in nuclear DNA, as our method compares the shortest possible extinction times for all corresponding selection coefficients (stronger selection on mtDNA than assumed will lead to even longer extinction times).

### C. briggsae

Populations of the sibling species of *C. elegans*, *C. briggsae*, also contain a very high fraction of self-fertilizing hermaphrodites. In many respects, *C. briggsae *shares similar life history characteristics with *C. elegans*, including levels of polymorphism and linkage disequilibrium, outcrossing rate, generation time, and fecundity [70]. Phenotypic assays of fitness in mutation accumulation lines suggest that *C. briggsae *might experience a higher mutation rate than *C. elegans *[59], although this observation has not yet been confirmed with direct mutation detection. *C. briggsae *shares a more recent common ancestor with the obligately outbreeding sibling species *C*. sp. 5 (JU727) than does *C. elegans *with any known sibling species, so in *C. briggsae *we can place a more recent upper bound on the time of persistence of self-fertilization (*T*_age_). Sequence divergence suggests that *T*_age _for *C. briggsae *is roughly 14 My [67], which implies that this species probably also experiences a genomic decay paradox with respect to Muller's ratchet in the same sense as *C. elegans*. A deeper sampling of species within the *Caenorhabditis *phylogeny will help elucidate whether a predominantly selfing mode of reproduction in *C. elegans *and *C. briggsae *arose recently in their evolutionary history.

## Conclusion

We find that *C. elegans *life history characteristics and evolutionary parameters are inconsistent with long-term survival as a complete selfer. Therefore, *C. elegans' *highly selfing lifestyle likely evolved relatively recently in evolutionary time. Alternatively, outcrossing and other factors have played a significant role in maintaining a tolerable mutation load throughout its history. If deleterious mutations are accumulating by Muller's ratchet and *C. elegans *is on its way to extinction, then the potential for compromised molecular biological phenomena should be taken into consideration in the analysis of *C. elegans *genetics and development.

## Methods

### Modeling extinction time due to Muller's Ratchet

We use the standard model of Muller's ratchet as described in Loewe [6] to compute *T*_*cl*_, the time between two clicks of the ratchet, from the effective population size *N*_*e*_, the genomic deleterious mutation rate *U*_*SDM*_, and the selection coefficient against deleterious mutations *s *(positive *s *denote deleterious mutations). To account for weaknesses in the various methods of computation, we combine some of the best analytic approximations available [19-21,6] with individual-based simulations that were computed on the evolution@home global computing network [6,22]. We also rely on the theoretical work described elsewhere [10-12] to apply these results that were computed for asexuals to selfers. In short, this means doubling selection coefficients (*s *→ 2*s*) to account for the improved power of selection in selfers and including deleterious mutations from the whole diploid genome (double mutation rate; *U*_*SDM *_and *s *increases cancel out). This makes the speed of Muller's ratchet in diploid selfers equivalent to the speed in asexual haploids. To account for the presence of a distribution of deleterious mutational effects, we use the appropriate approach as described elsewhere [12,71]. Briefly, we estimate *N*_*e *_under background selection from diversity data and then use exactly this *N*_*e *_for our simulation, as it has been shown to be appropriate for simulating Muller's ratchet in the presence of background selection without explicitly simulating background selection [12,71]. We ignore the long-term effects of effectively neutral mutations, as their combined potential for decreasing fitness is too small to be of interest here. Then we scale the total genomic mutation rate *U*_*TOT *_by *f*_*SDM*_, the fraction of slightly deleterious mutations that have critical selection coefficients as determined in Figures 1 and 2 to obtain *U*_*SDM*_. To compute extinction times, we first compute *C*_*mm*_, the number of clicks that are needed for mutational meltdown from *C*_*mm *_= log(1/*R*_*max*_)/log (1 - *s*), where *R*_*max *_is the maximal reproductive capacity, *s *is positive for deleterious mutations and fitness is assumed to be multiplicative as in Loewe [6]. The extinction time is then approximated by *T*_*ex *_= *C*_mm _* *T*_cl _* *T*_*gen*_, where *T*_*gen *_is the absolute generation time.

### C. elegans life history and evolutionary parameters

In order to calibrate the models and simulations, we collected relevant life history and evolutionary parameters from the literature. Based on these parameter ranges, we applied "best estimates" along with minimum and maximum values (Table 1) to the analytical and simulation models of Muller's ratchet to calculate extinction time. Potential nuclear and mitochondrial deleterious mutation rates span a wide range. To avoid overly crowded plots, we included one range of values along with our most plausible haploid nuclear deleterious mutation rate (= 0.5, Figure 1) and another range of values with our most plausible mitochondrial deleterious mutation rate (= 0.001, Figure 2).

**Table 1:** Assumed parameter values for *C. elegans *as applied to Muller's ratchet models of extinction time

Parameter	Best estimate	Minimum	Maximum
*T*_*gen*_	60 d	4 d	90 d
*R*_*max*_	280	100	1000
*T*_*age*_	18 Myr	5 Myr	30 Myr
*N*_*e*(*nuclear*)_	10000	1000	100000
*U*_*TOT *(nuclear, haploid)_	1.5	0.6	3
*U*_*SDM *(nuclear, haploid)_	0.5	0.06	1.5
*N*_*e *(mt)_	10000	1000	100000
*U*_*TOT *(mt)_	0.001	0.0005	0.002
*U*_*SDM *(mt)_	0.0003	0.00005	0.001
*f*_*SDM*_	30%	10%	50%
*s*			Many orders of magnitude (10^-6 ^- 1)

#### Population size

Effective population size (*N*_e_) in *C. elegans *has been estimated from nuclear nucleotide, microsatellite, and amplified fragment length polymorphism (AFLP) diversities to range between 1,000 and 100,000 [13,16,15]. The *N*_e _of mtDNA is expected to be equal to the nuclear value in pure selfers [72]. This equalized *N*_e _of mitochondrion and nucleus, relative to the 4:1 expectation for a gonochoristic or dioecious species, is due to (i) a doubling of the mitochondrial effective size because all hermaphrodites can pass mtDNA to their offspring (i.e., lack of males) and (ii) a halving of the nuclear effective size from selfing-induced homozygosity [73,74]. These estimates can be used directly in our simulations, as they are obtained under background selection [71].

#### Maximal reproductive capacity (*R_max_*)

The reproductive output of selfing *C. elegans *is limited by sperm production [75], and wild isolates vary relatively little in this quantity [76]. Different strains of *C. elegans *produce from an average of 187 to 353 self-progeny [76], although mated individuals are capable of producing up to 1000 offspring [77].

#### Generation length (*T_gen_*)

The generation time of *C. elegans *in nature is the least well-characterized parameter necessary for the extinction time models. The observation that most animals isolated from nature are found in the "enduring" dauer larval stage suggests that a 2 day generation time with *ad libidum *food conditions in the laboratory at 25°C is not typical of natural conditions. Dauer larvae can survive up to many months [77] and mutations exist that extend the lifetime of *C. elegans *considerably [78,79]. However, *C. elegans *dauer larvae are unlikely to be able to survive the long periods that anhydrobiotic species are capable of [80], because of their unsheathed cuticle [81]. Thus a mean generation time of 30 to 60 days may not be unreasonable. Furthermore, the lack of a long-lived dauer "seed bank" in *C. elegans *implies that populations cannot be reconstituted by quiescent stage larvae with a low mutation load.

#### Mutation rates

Estimates of the deleterious mutation rate (*U*_*SDM*_) in *C. elegans *have been calculated from observable phenotypic declines in fitness in laboratory mutation accumulation lines [82-84] as well as from the direct detection of sequence changes in some of the same mutation accumulation lines [85,86]. Phenotypic estimates of *U*_*SDM *_have yielded values between 0.0026 and 0.025 deleterious mutations/haploid genome/generation [82-84]. However, Davies et al. [87] suggest that these measures should be adjusted upward 28-fold because approximately 96% of deleterious mutations are undetectable experimentally, a result that was corroborated with a different approach [48]. Such a correction leads to values of 0.073 and 0.7, respectively. Direct sequencing also suggests a value of *U*_*SDM *_that is about 30 times higher than the initial phenotypic estimate for the same lines [85]. We consider direct sequencing to be more reliable here, as phenotypic mutation accumulation misses many small effects [87,48]. These effects would have an impact on evolutionary timescales.

The total mutation rate *U*_*TOT *_at potentially deleterious sites can be computed from the observable per base pair mutation rate per generation, μ, and from the mutational target size *G*. For mitochondria μ_*mt *_= 1.6 × 10^-7 ^/bp/generation including indels and 8.9 × 10^-8 ^for point mutations only [88] with G ≈ 10 Kbp [89]. For nuclear sites μ_*nu *_= 2.1 × 10^-8 ^/bp/generation including indels and 9.1 × 10^-9 ^for point mutations only [85]. There are ≈ 26 Mbp in all exons of *C. elegans *[90]; this value has to be corrected, since we are not interested in synonymous sites and there are probably about three times more functional sites in noncoding DNA than in coding DNA [91-94], suggesting a total of G ≈ 70 Mbp functional sites per haploid genome. This accords with recent assessments that ~70% of the genome is functional [95]. Thus for the mitochondrial genome *U*_*TOT *_≈ 0.001/generation and for the haploid nuclear genome *U*_*TOT *_≈ 1.5/generation (including indels) and *U*_*TOT *_≈ 0.64/generation (point mutations only).

#### Distribution of deleterious mutational effects on fitness (DDME)

To estimate *U*_*SDM *_we have to consider (i) the total mutation rate *U*_*TOT *_which is given by the frequency of molecular changes of the DNA and (ii) the corresponding DDME. Information about the DDME is important, because Muller's ratchet would not operate if deleterious mutations all had effects large enough to be eliminated deterministically by selection. Alternatively, mutations could accumulate by Muller's ratchet very rapidly, yet have no effect on fitness, if all mutational effects were sufficiently small. Consequently, we consider extinction time as a function of the strength of selection to identify the range of critical selection coefficients *s*_*c *_that could lead to extinction in a given time interval. We then integrate over all mutational effects to estimate *f*_*SDM*_, the fraction of mutations that have deleterious effects in the range given by *s*_*c*_, so that *U*_*SDM *_= *U*_TOT _* *f*_*SDM*_. Although the DDME is largely uncharacterized in *C. elegans*, we assume here that a substantial proportion of mutational effects are distributed among all the orders of magnitude between lethality (*s *= 1) and *s *= 10^-6^. This assumption appears to be valid in Drosophila, and is likely to be a reasonable approximation for most taxa [23,24]. Figure 3 can be interpreted as to support this assumption, which uses the calculations of the nonsynonymous to synonymous site divergence ratio (*K*_*A*_/*K*_*S*_) between *C. elegans *and *C. briggsae *from Cutter & Ward [96]. Using this approach it is difficult to see how any particular critical selection coefficients could have *f*_*SDM *_< 10% or *f*_*SDM *_> 50% of *U*_*TOT*_. Therefore, these assumptions about *U*_*SDM *_appear to be justified biologically. This approach is robust to the complicating effects of background selection [71,97].

We also derived a rough point estimate of the DDME in *Caenorhabditis *using a method that requires only estimates of replacement (π_a_) and synonymous-site (π_s_) diversity for two species [23,50]. We used estimates of polymorphism for *C. elegans *and *C. remanei*. For 40 X-linked loci in *C. remanei *we use an average π_a _= 0.00102 and π_s _= 0.0350 [52]. Comparable measures for *C. elegans *are more difficult to obtain, because most estimates of nucleotide diversity identify too few replacement site polymorphisms to accurately estimate π_a_. Heuristically, we use the data of Koch et al. [51] for this purpose, assuming that their shotgun sequencing randomly sampled the genome with respect to coding and noncoding sequences and that no sequences from each of the 4 strains they sampled overlapped with each other; thus providing a set of single pairwise comparisons with the N2 strain. Using this logic, we compute π_a_~0.000155 and π_s _~ 0.000527 (22 replacement polymorphisms in 1.42 × 10^5 ^nonsynonymous basepairs, 25 synonymous polymorphisms in 4.7 × 10^4 ^synonymous basepairs). The total number of synonymous and nonsynonymous basepairs was calculated assuming that 26% of the 730 kb sequenced was exonic and that 25% of exonic sites are synonymous, as in the genomic average. We caution that these estimates for *C. elegans *are not ideal, although we expect that the relative magnitude of π_a _to π_s _is reasonable.

Using an approach described elsewhere [23,50], briefly, we fit lognormal DDME location and shape parameters to the levels of diversity observed for both types of sites in both species. For the above diversity values, μ_g _= 3.86 × 10^-4 ^and σ_g _= 15.76, where μ_g _is the geometric mean mutation rate and σ_g _is the geometric standard deviation. These parameters reflect median central tendency and allow easy computation of the boundaries that include 68% of all probability mass (lower limit = μ_g_/σ_g_; upper limit = μ_g _* σ_g_; μ_g _= exp [arithmetic mean on the log-scale] = median; σ_g _= exp [normal standard deviation on the log-scale]; see [98]). The resulting point estimate DDME (Figure 4) predicts credible frequencies of dominant lethal mutations if one assumes that *Caenorhabditis *and *Drosophila *are roughly similar in this respect [23]. Integrating the ranges of selection coefficients from 10^-2 ^to 0.1, 10^-3 ^to 0.1, and 10^-4 ^to 0.1 results in respective probability masses of *f*_*SDM *_= 9.7%, 34% and 66%. These calculations lend empirical support for the estimates compiled in Table 1.

### Phylogenetic context

In order to place the extinction time estimates into a biological context for *C. elegans*, we use inferences of intraspecific coalescent times within *C. elegans *and divergence times between *C. elegans *and its sister taxa. Using the expected coalescent time for a sample of segregating polymorphisms, Cutter [13] calculated that extant nucleotide polymorphism in this species likely coalesces roughly 60 Kya ago (= 4 *N*_*e *_generations). Maximum persistence times can be inferred from interspecific calculations of the time to the most recent common ancestor (*T*_*MRCA*_). Previous estimates of *T*_*MRCA *_for *C. elegans *and its relatives suggested that they diverged 80 – 110 Mya [69,68], although the use of non-nematode calibration of the molecular clock makes this divergence time almost surely a drastic overestimate [99,2]. Consequently, we prefer to use some recent dates of divergence using internal calibration [67], which suggest that *C. elegans *shared a most recent common ancestor with its outcrossing sister clade approximately 18 Mya (range 5 – 30 Mya). Use of this more recent divergence time is conservative for our purposes as it minimizes the potential of falsely inferring that the expected extinction time is shorter than lineage age.

## Authors' contributions

LL conceived the project and performed the computations. LL and ADC wrote the manuscript.
